# Predictors of aneurysm shrinkage after flow diversion treatment for internal carotid artery aneurysms: quantitative volume analysis with MRI

**DOI:** 10.3389/fneur.2023.1266460

**Published:** 2023-12-21

**Authors:** Ryo Akiyama, Akira Ishii, Takayuki Kikuchi, Masakazu Okawa, Yukihiro Yamao, Yu Abekura, Isao Ono, Natsuhi Sasaki, Hirofumi Tsuji, So Matsukawa, Susumu Miyamoto

**Affiliations:** ^1^Department of Neurosurgery, Kyoto University Graduate School of Medicine, Kyoto, Japan; ^2^Department of Neurosurgery, Hikone Municipal Hospital, Hikone, Japan

**Keywords:** flow diversion, aneurysm, shrinkage, MRI, volume analysis

## Abstract

**Background and purpose:**

Although aneurysm shrinkage often occurs after flow diversion treatment for intracranial aneurysms, no reports have addressed the factors associated with aneurysm shrinkage.

**Materials and methods:**

This retrospective single-center study was performed to examine patients with unruptured internal carotid artery aneurysms who were treated using flow diversion and followed up by imaging for at least 12 months. The study outcome was aneurysm shrinkage (volume reduction of ≥10%) 12 months after treatment. Aneurysm volume was quantitatively assessed using the MRIcroGL software. Patient and aneurysm characteristics were statistically analyzed.

**Results:**

This study involved 81 patients with 88 aneurysms. At the 6 months, 12 months, and last follow-ups, the proportion of aneurysms that had shrunk was 50, 64, and 65%, respectively. No adjunctive coiling (odds ratio, 56.7; 95% confidence interval, 7.03–457.21; *p* < 0.001) and aneurysm occlusion (odds ratio, 90.7; 95% confidence interval, 8.32–988.66; *p* < 0.001) were significantly associated with aneurysm shrinkage. In patients treated by flow diversion with adjunctive coiling, only the volume embolization rate was a factor significantly associated with aneurysm shrinkage (*p* < 0.001). Its cutoff value was 15.5% according to the receiver operating characteristic curve analysis (area under the curve, 0.87; sensitivity, 0.87; specificity, 0.83).

**Conclusion:**

The rate of aneurysm shrinkage after flow diversion increased during the first 12 months after treatment, but not thereafter. No adjunctive coiling and aneurysm occlusion were predictors of aneurysm shrinkage, respectively. If adjunctive coiling is required, a volume embolization rate of ≤15.5% may be suggested for aneurysm regression.

## Introduction

In recent years, the advent of the flow diverter (FD) has markedly improved the results of endovascular treatment of unruptured large/giant cerebral aneurysms (1, 2). Flow diversion treatment is being indicated for increasingly more types of aneurysms, and FDs have become indispensable devices in the endovascular treatment of cerebral aneurysms (3). FDs are designed to be placed across the aneurysmal neck to reduce blood flow within the aneurysmal sac, thus inducing progressive thrombosis and subsequent occlusion. Cerebral aneurysms treated with flow diversion often shrink over time by regression change (4–6). Previous studies showed that aneurysm shrinkage was associated with symptomatic improvement of cerebral aneurysms presenting with cranial neuropathy after flow diversion treatment (7). Histopathological studies in animal models and human autopsies suggest that aneurysm sac shrinkage after FD treatment is caused by intra-aneurysmal thrombus organization and retraction after neointimal coverage of the FD surface at the aneurysm neck. Therefore, aneurysm shrinkage is significant not only because it relieves the mass effect on important surrounding structures, but also because it implies histopathologic repair of the aneurysm (8–10). However, although several reports have evaluated such shrinkage qualitatively, few reports have evaluated it quantitatively (11–13). Moreover, no reports so far have identified the predictors of aneurysm shrinkage yet. Adjunctive coiling with flow diversion is often used for large intradural aneurysms with intent to reduce the risk of delayed aneurysm rupture. However, it remains unclear whether coil mass in the aneurysm disturbs aneurysm shrinkage.

This study was performed to quantitatively evaluate the course of aneurysm shrinkage and investigate predictors of aneurysm shrinkage after flow diversion treatment by closely following up imaging studies of aneurysms over a long period.

## Materials and methods

### Ethics approval

This retrospective single-center study was approved by our institutional review board. Informed consent was obtained using an opt-out method on the institutional website.

### Data collection

All patients who undergo flow diversion treatment of unruptured aneurysms in our institution are registered in a prospectively maintained database. Data for the period from April 2016 to March 2021 were retrospectively reviewed. Patients who were treated for an internal carotid artery (ICA) aneurysm with flow diversion and were followed up for at least 12 months were eligible for inclusion. Clinical data were obtained from the database and the patient’s medical records.

### Treatment strategy and endovascular procedure

Patients received 100 mg/day of aspirin and 75 mg/day of clopidogrel for 14 days before the procedure. Platelet function was routinely tested using the VerifyNow P2Y12 assay and the VerifyNow Aspirin assay (Accumetrics, San Diego, CA, United States) the day before the procedure. Antiplatelet medications were adjusted accordingly, as in previous reports (14). Dual antiplatelet therapy was continued for at least 6 months after the procedure, and single antiplatelet therapy was continued indefinitely thereafter.

All procedures were performed under general anesthesia using the standard transfemoral approach. Heparin anticoagulation was implemented throughout the procedure. Flow diversion treatment was performed using a standard technique, as described previously (14, 15).

FD implantation was performed by a neuroendovascular specialist with more than 10 years of experience in intracranial stent placement. The FD type and number were selected by the operator.

In principle, adjunctive coil embolization was performed if the aneurysm was located in the subarachnoid space to prevent delayed rupture. The volume embolization rate (VER), defined as the ratio of the volume of the packed coils to the aneurysm volume, was calculated in patients who underwent adjunctive coiling. The aneurysm volumes used to calculate the VER were determined by adapting the three-dimensional diameters from the three-dimensional rotational angiography data of the aneurysm to the following formula:


$$\mathrm{Aneurysm}\;\mathrm{volume}=D1\times D2\times D3\times \frac{\pi }{6}$$

### Quantitative volume assessment on MRI

**Figure 1:**
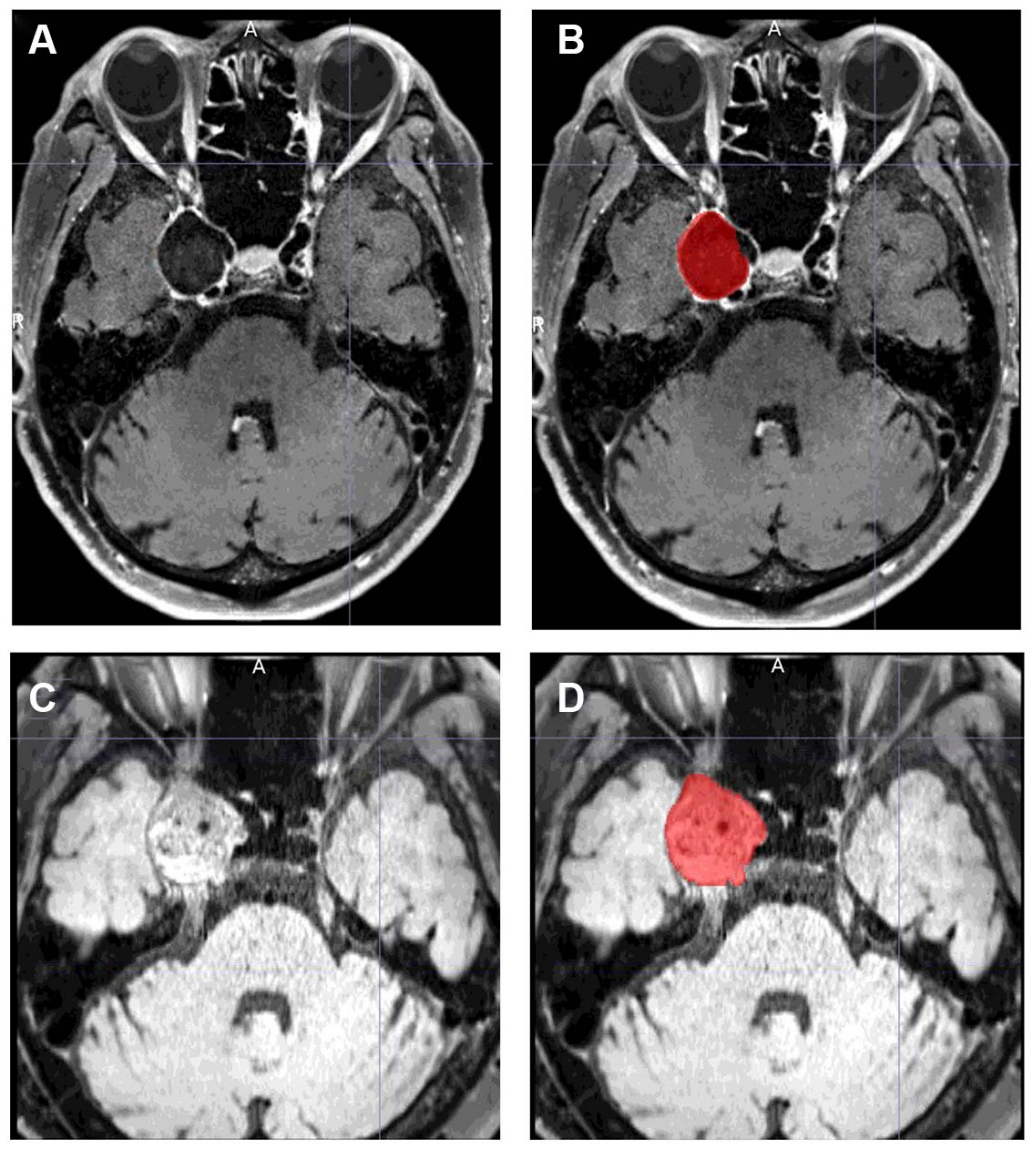
Examples of aneurysm segmentation using MRIcroGL software. **(A,C)** show images before segmentation, and **(B,D)** show images after segmentation. **(A,B)** is an example of aneurysm segmentation before treatment. The volume of the aneurysm is automatically calculated by manually segmenting all slices in which the aneurysm is present. DANTE T1-SPACE has high spatial resolution and can reliably identify the margins of an aneurysm. **(C,D)** is an example of aneurysm segmentation 12 months after treatment. Even if the aneurysm is thrombosed and shows a high signal thrombus on T1, the parent artery is black, making it easy to isolate and identify the aneurysm.

The angiographic outcome was assessed with digital subtraction angiography or magnetic resonance angiography 6 and 12 months after the procedure. Thereafter, imaging follow-up was continued every 6 to 12 months at the discretion of the operator. Other magnetic resonance imaging (MRI) sequences were also routinely performed: T1-weighted imaging, contrast-enhanced T1-weighted imaging, T2-weighted imaging, fluid-attenuated inversion recovery, T2*, diffusion-weighted imaging, and delay alternating with nutation for tailored excitation-prepared T1-weighted variable flip angle turbo spin echo (DANTE T1-SPACE) with and without contrast medium. These sequences were performed using a 3.0-Tesla MRI scanner (MAGNETOM Skyra; Siemens Healthineers, Erlangen, Germany) with a 32-channel head coil. Detailed imaging parameters are described in previous reports (16, 17). Aneurysm occlusion was categorized by the neuroendovascular specialist according to the O’Kelly–Marotta (OKM) grading scale (18). The aneurysm volume was assessed 6 and 12 months after the procedure and at the last follow-up using DANTE T1-SPACE. We used this MRI sequence because DANTE T1-SPACE has unprecedented spatial resolution, flow suppression, and artifact immunity, and we found it useful for aneurysm segmentation (19, 20). If this MRI sequence was not available, another sequence was used. Aneurysm volumes in all patients were measured with MRIcroGL software.^1^ Using this software, the segmentation of the aneurysm was manually drawn from each slice, and the aneurysm volumes were then calculated semi-automatically (Figure 1). The aneurysm volume variation rate was calculated using the following formula:


$$\begin{array}{l} \mathrm{Aneurysm}\;\\ \mathrm{volume variation rate}=100\times \frac{\begin{array}{l} \mathrm{Preoperative aneurysm volume}-\\ \mathrm{Postoperative aneurysm volume} \end{array}}{\mathrm{Preoperative aneurysm volume}} \end{array}$$

Aneurysm volume variations were described as collapsed, shrunk, enlarged, or stable. Collapsed was defined as a variation rate of ≥50%, shrunk as ≥10%, and enlarged as ≤−10%. We determined these cut-off values based on previous literature (11, 13). Aneurysm volume variation of <10% and >−10% was defined as stable. Since the expected volume reduction rate would be less in the coiled aneurysms because of the inevitable volume of the coil mass, we calculated the aneurysm volume variation rate with coil volume correction for cases with adjunctive coil embolization using the following formula (Pre: preoperative aneurysm volume, Post: postoperative aneurysm volume):


$$\begin{array}{l} \mathrm{Aneurysm volume}\;\\ \mathrm{variation ratio with}\;\\ \mathrm{coil volume correction}=100\times \frac{\left(\begin{array}{l} Pre-\\ \mathrm{inserted}\;\\ \mathrm{coil volume} \end{array}\right)-\left(\begin{array}{l} \mathrm{Post}-\\ \mathrm{inserted}\;\\ \mathrm{coil volume} \end{array}\right)}{Pre-\mathrm{inserted coil volume}} \end{array}$$

One author measured the aneurysm volume while blinded to the patient’s clinical and aneurysm information and angiographical outcomes. To evaluate interobserver variability of assessment of the aneurysm volume variation 12 months after treatment, another author measured the aneurysm volume of the first 42 aneurysms.

### Clinical assessment and outcome

In patients who presented with cranial neuropathy due to aneurysms, symptom improvement was assessed 12 months after the procedure. Neurological assessment was performed by an examiner using the same symptom scale before and after treatment to ensure consistency.

The study outcome was aneurysm shrinkage 12 months after flow diversion treatment.

### Statistical analyses

Statistical analyses were performed using JMP Pro software version 16 (SAS Institute, Cary, NC, United States). Continuous data are presented as median with interquartile range (IQR) and were compared using the Mann–Whitney U test or Kruskal–Wallis test. The normality of continuous data was checked by the Shapiro–Wilk test. Categorical data are presented as numbers with percentages and were compared using Fisher’s exact test. Variables found to be significantly associated with aneurysm shrinkage in the univariate analysis were further evaluated using multivariable logistic regression. The predictive power of each parameter was evaluated by creating a receiver-operating characteristic (ROC) curve and calculating the area under the curve with the 95% confidence interval (CI). The optimal cut-off value of the continuous variable of the present data set was defined using ROC curve analysis. The relationship between the aneurysm volume variation rate and the VER was assessed using Spearman’s correlation coefficient. Interobserver variability of the assessment of aneurysm shrinkage 12 months after treatment between two observers was tested using *κ* statistics. A value of *p* of <0.05 was considered statistically significant.

## Results

### Patient and aneurysm characteristics

Eighty-one patients with 88 aneurysms met the inclusion criteria for the study. The patient and aneurysm characteristics are summarized in Table 1. The patients’ median age was 64 years (IQR, 52–73 years). Seventy-one patients (88%) were women and 10 (12%) were men. Comorbidities included hypertension in 42 patients (52%), dyslipidemia in 29 (36%), diabetes in 4 (5%), a history of smoking with a Brinkman index of >100 in 28 (35%), and obesity (defined as a body mass index >30 kg/m^2^) in 6 (7%). The aneurysm was located in the cavernous ICA in 32 patients (36%), paraclinoid ICA in 42 (48%), ICA-posterior communicating artery in 13 (15%), and ICA-anterior choroidal artery in 1 (1%). The median maximum aneurysm diameter was 11.9 mm (IQR, 10.0–19.4 mm). The median neck size was 6.4 mm (IQR, 4.8–8.1 mm). The median pretreatment aneurysm volume was 670 mm^3^ (IQR, 243–2,595 mm^3^). Forty (45%) aneurysms were symptomatic and 15 (17%) were partially thrombosed. The mean number of FDs implanted per patient was 1.2 (range, 1–5); multiple FDs were implanted for 11 aneurysms (13%). The FDs implanted were the Pipeline embolization device (Medtronic Neurovascular, Irvine, CA, United States) for 83 aneurysms (94%) and the Flow Redirection Endoluminal Device (MicroVention, Aliso Viejo, CA, United States) for 5 (6%). Adjunctive coiling was performed for 49 aneurysms (56%), all of which were located in the subarachnoid space. The median VER in the 49 patients who underwent coiling was 16.6% (IQR, 11.7–22.2%).

**Table 1:** Patient and aneurysm characteristics.

No of patients	*n* = 81
No of aneurysms	*n* = 88
Age (*y*)	64 (52–73)
Women	71 (88)
Comorbidities	
Hypertension	42 (52)
Dyslipidemia	29 (36)
Diabetes mellitus	4 (5)
History of smoking	28 (35)
Obesity	6 (7)
Aneurysm characteristics	
Aneurysm size (mm)	11.9 (10.0–19.4)
Aneurysm neck (mm)	6.4 (4.8–8.1)
Pre-treatment aneurysm volume (mm^3^)	670 (243–2,595)
Aneurysm location	
Cavernous portion	32 (36)
Paraclinoid portion	42 (48)
ICA-PC	13 (15)
ICA-Ach	1 (1)
Symptomatic aneurysm	40 (45)
Thrombosed aneurysm	15 (17)
Procedure characteristics	
Multiple stents used	11 (13)
Adjunctive coiling	49 (56)
VER (%)	16.6 (11.7–22.2)
MRI follow-up (mo.)	36.5 (22.5–51.5)
Aneurysm occlusion at 6 mo.	
OKM grade: D	45 (51)
OKM grade: C-D	69 (78)
Aneurysm occlusion at 12 mo.	
OKM grade: D	61 (69)
OKM grade: C-D	76 (86)
Morbidity	
Symptomatic hemorrhagic stroke	1 (1)
Symptomatic ischemic stroke	2 (2)
30-day major stroke	1 (1)
Re-treatment	8 (9)

Values shown are median (interquartile range) or number (percentage). ICA-PC, internal carotid artery-posterior communicating artery; ICA-Ach, internal carotid artery-anterior choroidal artery; VER, volume embolization rate; OKM, O’Kelly–Marotta.

### Angiographic follow-up

The rate of OKM grade D occlusion (no aneurysm filling) at 6 and 12 months was 51 and 69%, respectively. The rate of OKM grade C (small neck remnant) or D occlusion at the same time points was 78 and 86%, respectively.

### Complications and retreatment

Ipsilateral symptomatic intracerebral hemorrhage occurred as a complication in one patient (2%). Ipsilateral symptomatic embolic ischemic cerebral infarction occurred in another (2%). Major stroke, defined as deterioration in the modified Rankin scale score by ≥2 points, occurred in one patient in the first 30 days after treatment.

Retreatment was performed in eight patients (9%) at a median of 22.5 months (IQR, 12.3–25.8 months) after the initial procedure; all but one were retreated more than 1 year later. Retreatment consisted of overlapping the same type of FD used in the initial treatment; in one patient, however, overlapping did not occlude the aneurysm, and parent artery occlusion was therefore performed.

### Aneurysm volume and clinical assessment

**Figure 2:**
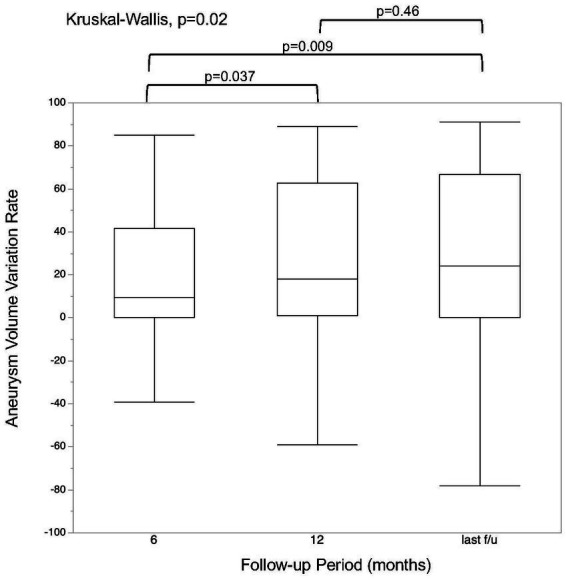
Regression rate at each follow-up time point. The last follow-up was performed at a median of 36.5 months (interquartile range, 22.5–51.5 months).

**Figure 3:**
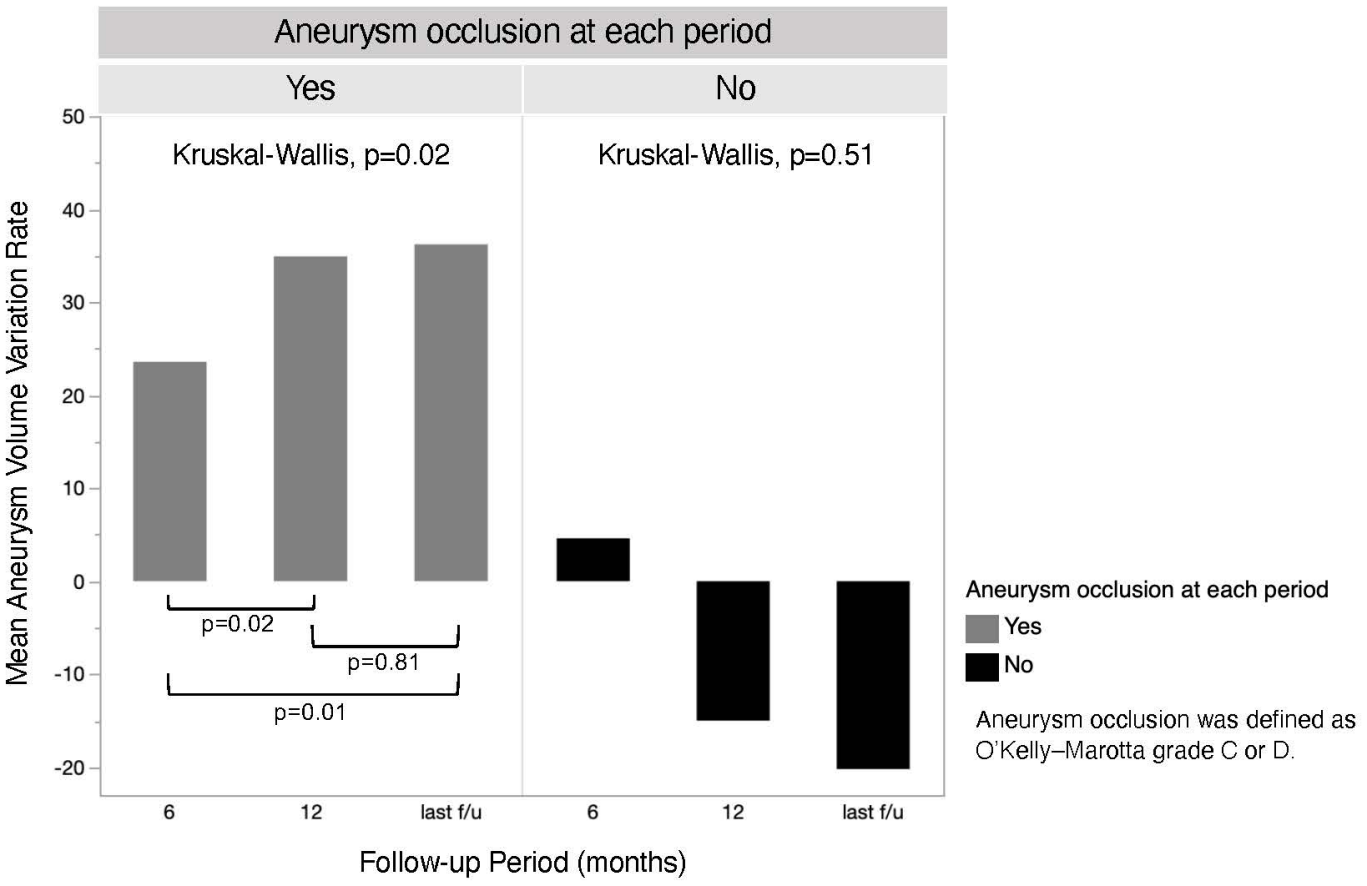
The chronological aneurysm volume variation rate of both aneurysm occluded and non-occluded groups. The last follow-up was performed at a mean of 36.7 months (standard deviation, ±17.1 months) in the occluded group and 39.7 months (standard deviation, ±22.3 months) in the non-occluded group.

MRI follow-up was performed at a median of 36.5 months (IQR, 22.5–51.5 months). The aneurysm volume variation rate at the 6 month, 12 month, and last follow-up is shown in Figure 2. There was a significant difference in the aneurysm volume variation rate between 6 and 12 months (*p* = 0.04), but not between 12 months and the last follow-up (*p* = 0.46). The aneurysm volume variation rate over time, divided into aneurysm occluded and non-occluded cases, is shown in Figure 3. In the occluded group, there was a trend toward a decrease in aneurysm size until 12 months, whereas, in the non-occluded group, there was no statistically significant difference between each period.

The degree of agreement between the two independent observers was good for aneurysm shrinkage 12 months after flow diversion treatment (*κ* = 0.80; 95% CI, 0.58–1.00).

**Figure 4:**
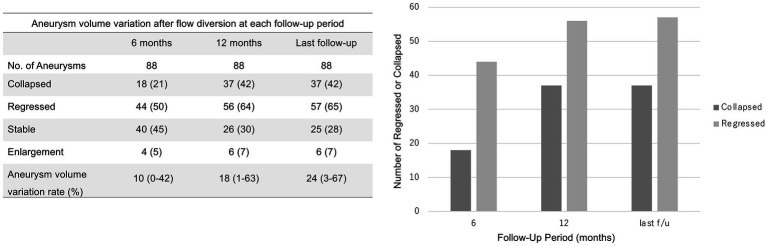
Aneurysm volume variation 6 and 12 months after treatment and at last follow-up. The last follow-up occurred at a median of 36.5 months (interquartile range, 22.5–51.5 months). The values shown are numbers (percentage).

**Figure 5:**
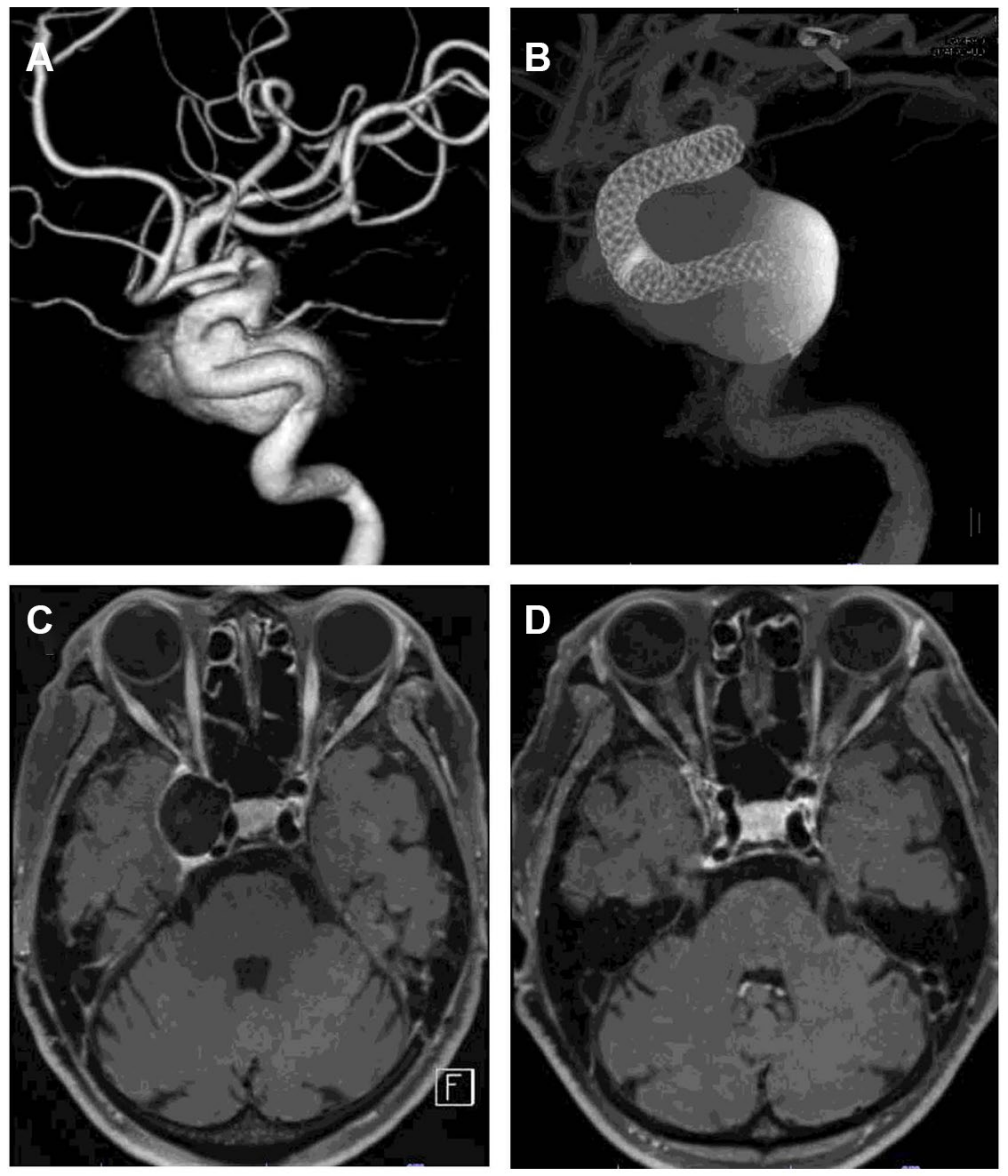
Representative case of aneurysm shrinkage after flow diversion treatment. **(A)** is a 3D rotational angiogram of the aneurysm. The aneurysm was located in the right cavernous portion with a maximum diameter of 23 mm. The patient had diplopia. The aneurysm was treated with two flow diverters **(B)**. **(C,D)** are DANTE T1-SPACE images before and 12 months after treatment. Twelve months after treatment, the aneurysm was significantly shrunk (89% reduction rate). DSA 12 months after treatment showed that the aneurysm was completely occluded, and the patient’s diplopia had disappeared.

**Figure 6:**
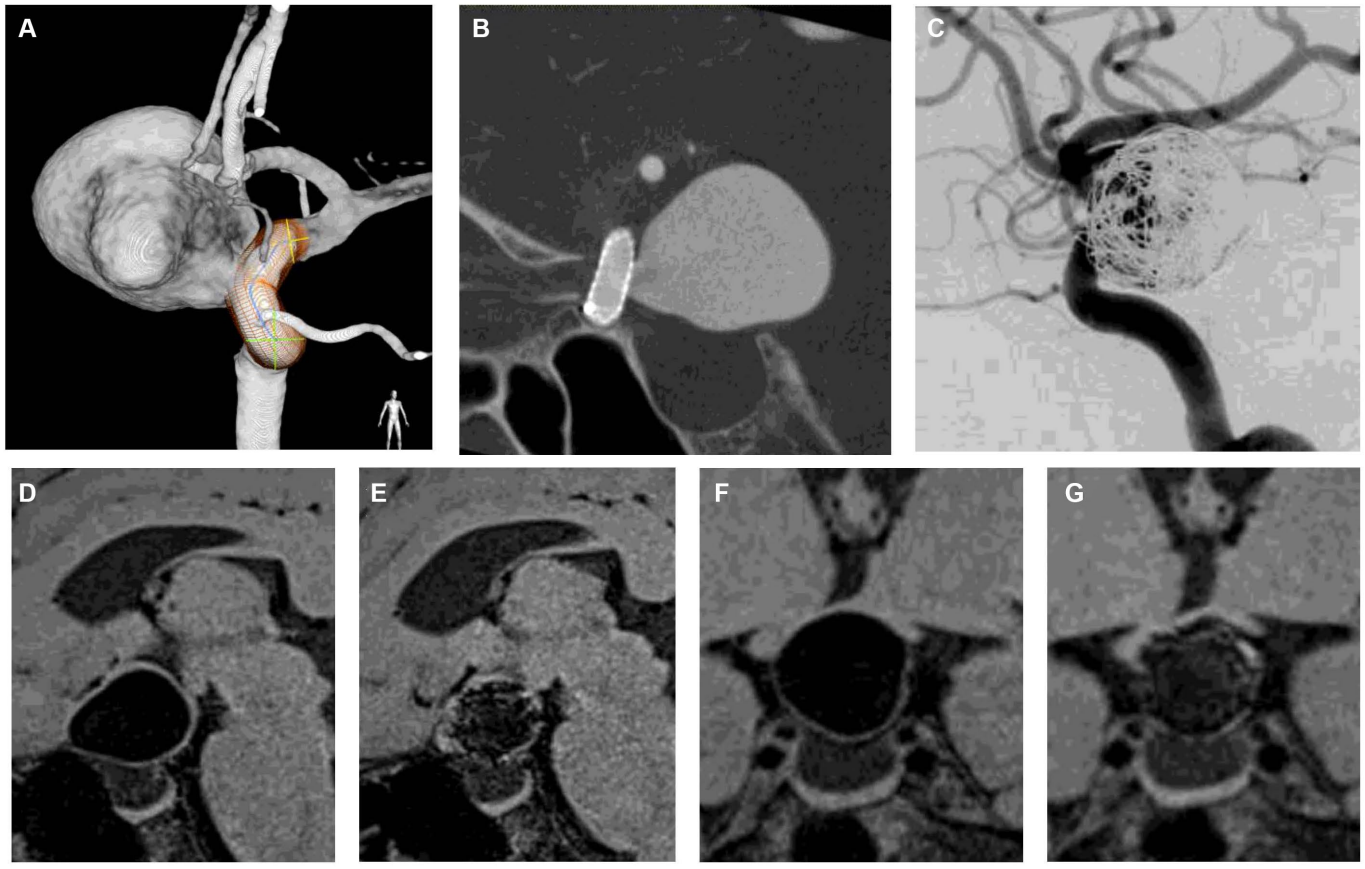
Representative case of aneurysm shrinkage after flow diversion with loose coil embolization. **(A)** is a 3D rotational angiogram of the aneurysm. The aneurysm was located in the paraclinoid portion with a maximum diameter of 23 mm. The aneurysm was compressing the optic nerve, and the patient had visual field defects. **(B)** is a corn beam CT after flow diverter implantation. **(C)** shows DSA after adjunctive coil embolization with a VER of 6.1%. **(D,F)** are DANTE T1-SPACE before treatment, and **(F,G)** are DANTE at 12 months after treatment. Twelve months after treatment, the aneurysm was shrunk (24% reduction rate), indicating that the optic nerve compression was relieved **(D–G)**. DSA 12 months after treatment showed that the aneurysm was completely occluded, and the patient’s visual field defects had improved. DANTE T1-SPACE has high artifact immunity and allows easy visualization of the aneurysmal margins even when the coil is implanted within the aneurysm **(E,G)**.

Figure 4 shows the course of aneurysm volume variation over time after treatment. At the 6 month, 12 month, and last follow-ups, the proportion of aneurysms that had shrunk was 50, 64, and 65%, respectively. At the 6 months, 12 months, and last follow-ups, the proportion of aneurysms that had collapse was 21, 42, and 42%, respectively. Representative cases are shown in Figures 5, 6.

The results of the aneurysm volume variation ratio with coil volume correction showed that the aneurysm volume variation rate was slightly higher in the group with coil volume correction than in the group without coil volume correction, but the difference was not statistically significant (Table 2). The number of shrunken aneurysms, defined as a volume reduction of ≥10%, was precisely the same at all time points for the coil-volume-corrected and non-coil-volume-corrected groups.

**Table 2:** Comparison of aneurysm volume variation ratio with and without coil volume correction in the group with adjunctive coil embolization.

Variables		Coil volume correction		*p* value
		Yes (*n* = 39)	No (*n* = 39)	
Mean aneurysm volume variation rate (%)	6 months	5 (±9)	4 (±8)	0.94
	12 months	9 (±0.15)	8 (±13)	0.77
	Last follow-up	8 (±14)	7 (±13)	0.98

Values shown are mean (± standard deviation).

Aneurysm enlargement occurred in six aneurysms (7%) 12 months after treatment. None of the enlarged aneurysms corresponded to OKM grade C or D at 6 and 12 months. The median size of the enlarged aneurysms was 22.9 mm (IQR, 14.0–25.6 mm), while that of the other aneurysms was 11.5 mm (IQR, 8.1–13.8 mm). The median neck size of the enlarged aneurysms was 9.3 mm (IQR, 4.7–7.7 mm), while that of the other aneurysms was 6.0 mm (IQR, 14.0–25.6 mm). Four (67%) of the enlarged aneurysms required retreatment.

Improvement of cranial neuropathy 12 months after flow diversion treatment was observed in 34 of 40 patients (85%).

### Outcome

Aneurysm shrinkage 12 months after flow diversion treatment was observed in 56 aneurysms (64%).

In the univariate analysis, aneurysm shrinkage was associated with cranial neuropathy improvement in patients with symptomatic aneurysms (odds ratio [OR], 7.7; 95% CI, 1.17–51.06; *p* = 0.04).

The results of the univariate and multivariate analyses of predictors of aneurysm shrinkage are summarized in Table 3. In the univariate analysis, factors significantly associated with aneurysm shrinkage were no adjunctive coiling (*p* < 0.001) and aneurysm occlusion at 12 months (*p* = 0.007). The multivariate analysis of these factors showed that no adjunctive coiling (OR, 56.7; 95% CI, 7.03–457.21; *p* < 0.001) and aneurysm occlusion at 12 months (OR, 90.7; 95% CI, 8.32–988.66; *p* < 0.001) remained independent predictors of aneurysm shrinkage after flow diversion treatment.

**Table 3:** Univariate and multivariate analyses of predictors of aneurysmal regression after flow diversion treatment.

Variable	Univariate			Multivariate		
	+ regression (*n* = 56)	- regression (*n* = 32)	*p* value	OR	95% CI	*p* value
Age (*y*)	65 (55–73)	59 (50–73)	0.34			
Women	49 (88)	27 (84)	0.75			
Hypertension	27 (48)	18 (56)	0.51			
Dyslipidemia	19 (34)	13 (41)	0.65			
Diabetes mellitus	2 (4)	2 (6)	0.62			
Smoker	21 (38)	11 (34)	0.82			
Obesity	3 (5)	4 (13)	0.25			
Aneurysm size (mm)	12.6 (10.0–19.4)	10.9 (8.2–19.3)	0.17			
Aneurysm neck (mm)	6.7 (4.9–8.3)	5.5 (4.4–8.1)	0.13			
Thrombosed aneurysm	11 (20)	4 (13)	0.56			
No adjunctive coiling	41 (73)	8 (25)	<0.001	56.7	7.03–457.21	<0.001
Aneurysm occlusion*						
6 months	47 (84)	22 (69)	0.11			
12 months	53 (95)	23 (72)	0.007	90.7	8.32–988.66	<0.001

Values shown are median (interquartile range) or number (percentage). ^*^Aneurysm occlusion was defined as O’Kelly–Marotta grade C or D. OR, odds ratio; CI, confidence interval.

**Figure 7:**
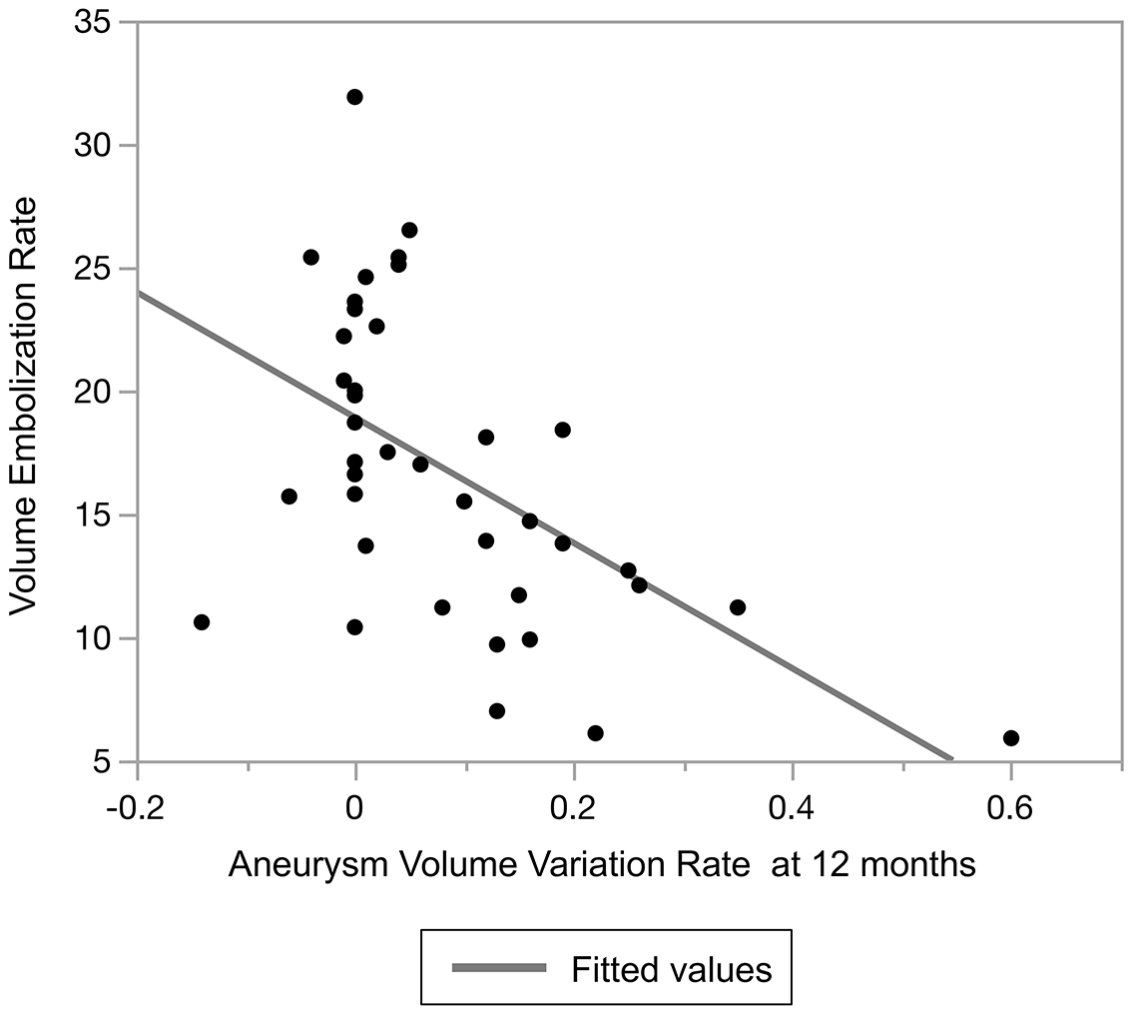
Relationship between volume embolization rate and aneurysm volume variation rate at 12 months after treatment.

Because of the variety of VERs observed in our cohort, we evaluated the relationship between the VER and aneurysm shrinkage using Spearman’s correlation coefficient. There was a statistically significant negative correlation between the VER and the aneurysm variation rate (rho = −0.527, *p* < 0.001) (Figure 7).

**Figure 8:**
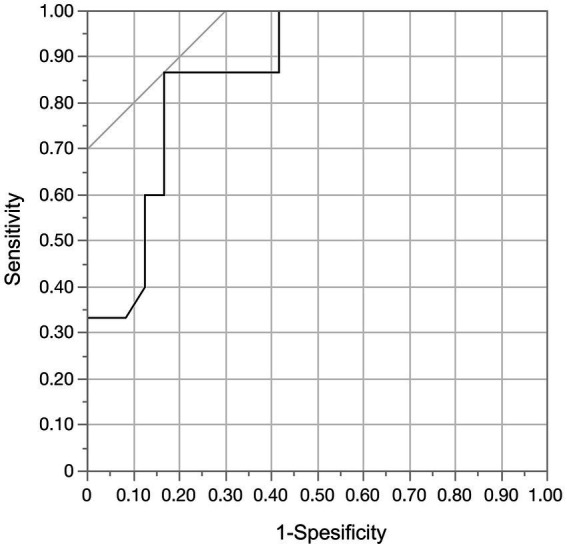
In the receiver operating characteristic curve analysis of aneurysmal regression and the volume embolization rate, the area under the curve was 0.868. With a cut-off value of 15.5%, the sensitivity was 0.87 and the specificity was 0.83.

Moreover, we analyzed predictors of aneurysm shrinkage in patients treated by flow diversion with adjunctive coiling to investigate whether the VERs were associated with aneurysm shrinkage. The results of the univariate analysis of predictors of aneurysm shrinkage are summarized in Table 4. Only the VER was a factor significantly associated with aneurysm shrinkage (*p* < 0.001). ROC curve analysis was performed to determine the optimal cut-off value of the VER for aneurysm shrinkage. The area under the ROC curve was 0.87, and the sensitivity and specificity were 0.87 and 0.83, respectively, with a cutoff value of 15.5% (Figure 8). A VER of ≤15.5% was also found to be a predictor of aneurysm shrinkage (OR, 32.5; 95% CI, 5.19–203.7; *p* < 0.001).

**Table 4:** Univariate analysis of predictors of aneurysmal regression after flow diversion with coils.

Variables	+ regression (*n* = 15)	- regression (*n* = 24)	*p* value
Age (*y*)	55 (45–71)	56 (50–73)	0.69
Women	13 (87)	20 (83)	1.00
Hypertension	5 (33)	11 (46)	0.52
Dyslipidemia	3 (20)	9 (38)	0.31
Diabetes mellitus	0 (0)	1 (4)	1.00
History of smoking	9 (60)	11 (46)	0.52
Obesity	3 (20)	3 (13)	0.66
Aneurysm size (mm)	11.3 (8.0–15.0)	10.1 (7.8–13.3)	0.31
Aneurysm neck (mm)	5.9 (4.7–7.3)	4.9 (3.8–6.8)	0.22
Thrombosed aneurysm	0 (0)	1 (4)	1.00
VER	12.1 (9.7–14.7)	19.9 (16–24.4)	<0.001
VER ≤15.5	13 (87)	4 (17)	<0.001
Aneurysm occlusion*			
6 months	14 (93)	21 (88)	1.00
12 months	15 (100)	22 (92)	0.51

Values shown are median (interquartile range) or number (percentage). VER, volume embolization rate. ^*^Aneurysm occlusion was defined as O’Kelly–Marotta grade C or D.

## Discussion

To the best of our knowledge, this is the first study to investigate predictors of aneurysm shrinkage after flow diversion treatment in patients with ICA aneurysms. In addition, this is the largest study to quantitatively analyze the aneurysm volume after flow diversion. In our study, as in a previous report (12), the proportion of aneurysms that shrank increased during the first 12 months. After 12 months, however, this proportion did not change. This can be explained by aneurysm shrinkage being associated with aneurysm occlusion. Previous studies have shown that the aneurysm occlusion rate increases during the first 12 months after flow diversion treatment, but not much after (15, 21). We suspect that like the occlusion rate, the rate of aneurysm shrinkage does not markedly change after 12 months.

Previous studies reporting aneurysm shrinkage after flow diversion treatment are summarized in Table 5 (4–6, 11–13, 22–25). In the present study, 65% of aneurysms had shrunk at the last follow-up, which is a lower rate than in previous studies. This difference is due to the higher proportion of aneurysms with adjunctive coiling in our study than in previous studies. Because adjunctive coiling prevents aneurysm shrinkage, the proportion of shrunk aneurysms decreased as the performance of adjunctive coiling increased. In support of this, Carneiro et al. (11) reported a higher rate of adjunctive coiling (75%) and a lower rate of aneurysm shrinkage (28%). By contrast, Wang et al. (13) and Sirakova et al. (12) reported high aneurysm shrinkage rates of 76 and 83%, respectively, despite the high rate of adjunctive coiling. However, in these studies, all aneurysms with adjunctive coiling underwent loose coil embolization, which is thought to result in a better aneurysm shrinkage rate. In our study, the rate of aneurysm shrinkage was 88%, excluding cases with coils. Across all studies, the overall rate of aneurysm shrinkage after flow diversion was 77% (336/439).

**Table 5:** Previous studies reporting aneurysm shrinkage after flow diversion treatment.

References	Number of Aneurysm	Adjunctive coiling	Completely collapses	Decrease in size*	Unchanged in size	Increase in size	Follow-up period‡
Burge et al. (4)	66	9 (13.6%)	18 (27%)	53 (80%)	7 (11%)	6 (9%)	12
Piano et al. (5)	36	NA	22 (61%)	27 (75%)	8 (22%)	1 (3%)	12
Szikora et al. (22)	30	0 (0%)	27 (90%)	28 (97%)	1 (3%)	0 (0%)	18
Carneiro et al. (11)	8	6 (75%)	NA	3 (28%)	3 (38%)	2 (25%)	20
Slater et al. (23)	14	0 (0%)	2 (14%)	12 (86%)	0 (0%)	2 (14%)	24
Miyachi et al. (24)	19	0 (0%)	NA	17 (89%)	NA	NA	6
Patzig et al. (25)	25	0 (0%)	12 (48%)	19 (76%)	6 (24%)	0 (0%)	27
Wang et al. (13)	17	17 (100%)	NA	13 (76%)	4 (23%)	0 (0%)	25.5
Piano et al. (6)	100	NA	NA	78 (78%)	10 (10%)	2 (2%)	12–24
Sirakova et al. (12)	36	13 (36%)	NA	30 (83%)	6 (25%)	0 (0%)	12
Current study	88	49 (56%)	37 (42%)	56 (64%)	26 (30%)	6 (7%)	12
Total	439	–	118 (27%)	336 (77%)	72 (16%)	18 (4%)	–

^*^Decrease in size included completely collapsed aneurysms. ‡Follow-up period is expressed in months. NA, not available.

### Mechanism of aneurysm shrinkage

Aneurysms that have been occluded and thrombosed after flow diversion treatment are expected to shrink first by infiltration of inflammatory cells such as macrophages and then by deposition of a vascularized fibrous connective tissue scar (8–10, 26). Organization of the thrombus is considered to require endothelization of the aneurysm neck area covered by the FD (9). The lack of aneurysm shrinkage after flow diversion, even if the aneurysm is occluded, may indicate that the thrombus inside the aneurysm is not organized and that endothelialization of the aneurysm neck is not complete. In other words, aneurysms that do not shrink can be expected to induce thromboembolism upon discontinuation of antiplatelet medications or to recur due to exposure of unstable clots to the blood flow (27). Thus, in addition to its ability to relieve the mass effect on surrounding important structures, aneurysm shrinkage may be important in predicting histopathologic repair of aneurysms. The present study is meaningful in that it analyzed the predictors of aneurysm shrinkage after flow diversion.

### Factors related to aneurysm shrinkage

In the present study, no adjunctive coiling was associated with aneurysm shrinkage after flow diversion treatment. Analysis of only cases with adjunctive coiling showed that aneurysm shrinkage was more likely to be obtained with a lower VER, especially with a VER of ≤15.5%. In our previous study, aneurysm shrinkage was associated with symptomatic improvement of cerebral aneurysms presenting with cranial neuropathy after flow diversion treatment. Aneurysms located in the subarachnoid space are at risk for delayed rupture after flow diversion treatment, and adjunctive coiling may prevent this (28, 29). Because the presence of symptoms due to the aneurysm mass effect is a risk factor for delayed rupture, symptomatic aneurysms located in the subarachnoid space undergoing flow diversion treatment should also be coiled to prevent delayed rupture (28, 29). However, the adjunctive coiling may not result in shrinkage of the aneurysm, thereby deteriorating the prognosis for cranial neuropathy. A solution to this dilemma is to perform loose coil packing, although the most appropriate VER with such treatment is unknown. The results of the present study, in which coil embolization with a target VER of ≤15.5% did not prevent aneurysm shrinkage, may serve as an indicator to help resolve this issue. This index of a VER of ≤15.5% is relatively similar to the index of a VER of <13% presented by Akiyama et al. (7) and the index of a VER of <12% presented by Wang et al. (13), and it thus appears to be a reasonably reliable index.

Aneurysm occlusion was also associated with aneurysm shrinkage. Our study defined aneurysm occlusion as OKM grade C or D, and complete occlusion was not always necessary for aneurysm shrinkage.

We had expected that aneurysm thrombosis would be an inhibiting factor for aneurysm shrinkage because the thrombosed areas of the aneurysms would likely be organized and would not shrink. However, aneurysm thrombosis was not a significant factor in this study. Thrombosed aneurysms often shrank once occlusion of the aneurysm had been obtained. This may suggest that the thrombus within the thrombosed aneurysm was unorganized and that it subsequently became organized and shrank following aneurysm occlusion.

### Factors related to aneurysm enlargement

Although a statistical analysis was not possible because of the small number of enlarged aneurysms in the current study, aneurysm non-occlusion appeared to be associated with enlargement. In addition, enlarged aneurysms tended to have a larger size and wider neck than other aneurysms. Aneurysms that are expected to be difficult to occlude with flow diversion and aneurysms with a large size or broad neck should be treated with caution, and alternative treatment options may need to be considered.

### Limitations

This study has several limitations. Although prospectively collected data were used, the analyses were retrospective and thus had inherent limitations. Additionally, the aneurysm dome size and neck size had a small tendency to be associated with aneurysm shrinkage, but these factors were not included in the multivariate analysis because their inclusion could have reduced the quality of the analysis. Therefore, they may have been confounders. Finally, measurement errors might have occurred. However, because the inter-rater reliability between the two examiners was good, the error seemed to be within an acceptable range.

## Conclusion

The rate of aneurysm shrinkage after flow diversion increased during the first 12 months after treatment, but not thereafter. No adjunctive coiling and aneurysm occlusion were predictors of aneurysm shrinkage after flow diversion treatment. Aneurysm shrinkage may be achieved with a VER of ≤15.5% if adjunctive coiling is required.

## Data availability statement

The raw data supporting the conclusions of this article will be made available by the authors, without undue reservation.

## Ethics statement

The studies involving humans were approved by the Kyoto University Hospital Institutional Review Board (ID R0058). The studies were conducted in accordance with the local legislation and institutional requirements. Written informed consent for participation was not required from the participants or the participants’ legal guardians/next of kin because informed consent was obtained using an opt-out method on the institutional website.

## Author contributions

RA: Conceptualization, Data curation, Formal analysis, Investigation, Methodology, Validation, Visualization, Writing – original draft, Writing – review & editing. AI: Conceptualization, Data curation, Formal analysis, Investigation, Supervision, Validation, Writing – original draft, Writing – review & editing. TK: Data curation, Writing – review & editing. MO: Data curation, Writing – review & editing. YY: Data curation, Writing – review & editing. YA: Data curation, Writing – review & editing. IO: Data curation, Formal analysis, Writing – review & editing. NS: Data curation, Writing – review & editing. HT: Data curation, Writing – review & editing. SMa: Data curation, Writing – review & editing. SMi: Supervision, Writing – review & editing.

## Footnotes

^1^ http://www.mccauslandcenter.sc.edu/mricrogl/home

## References

[1] Kallmes DF Brinjikji W Cekirge S Fiorella D Hanel RA Jabbour P. Safety and efficacy of the pipeline embolization device for treatment of intracranial aneurysms: a pooled analysis of 3 large studies. J Neurosurg. (2017) 127:775–80. doi: 10.3171/2016.8.JNS16467, PMID: 27791519

[2] Becske T Brinjikji W Potts MB Kallmes DF Shapiro M Moran CJ. Long-term clinical and angiographic outcomes following pipeline embolization device treatment of complex internal carotid artery aneurysms: five-year results of the pipeline for uncoilable or failed aneurysms trial. Neurosurgery. (2017) 80:40–8. doi: 10.1093/neuros/nyw014 28362885

[3] Hanel RA Kallmes DF Lopes DK Nelson PK Siddiqui A Jabbour P. Prospective study on embolization of intracranial aneurysms with the pipeline device: the PREMIER study 1 year results. J NeuroIntervent Surg. (2020) 12:62–6. doi: 10.1136/neurintsurg-2019-015091, PMID: 31308197 PMC6996098

[4] Berge J Biondi A Machi P Brunel H Pierot L Gabrillargues J. Flow-diverter silk stent for the treatment of intracranial aneurysms: 1-year follow-up in a multicenter study. Am J Neuroradiol. (2012) 33:1150–5. doi: 10.3174/ajnr.A2907, PMID: 22300924 PMC8013262

[5] Piano M Valvassori L Quilici L Pero G Boccardi E. Midterm and long-term follow-up of cerebral aneurysms treated with flow diverter devices: a single-center experience; special topic. J Neurosurg. (2013) 118:408–16. doi: 10.3171/2012.10.JNS112222, PMID: 23176329

[6] Piano M Luca V Emilio L Guglielmo P Luca Q Edoardo B. FRED Italian registry: a multicenter experience with the flow re-direction Endoluminal device for intracranial aneurysms. J Neurosurg. (2019) 133:1–8. doi: 10.3171/2019.1.JNS183005, PMID: 31075778

[7] Akiyama R Ishii A Kikuchi T Okawa M Yukihiro Yamao Y. Onset-to-treatment time and aneurysmal regression predict improvement of cranial neuropathy after flow diversion treatment in patients with symptomatic internal carotid artery aneurysms. J NeuroIntervent Surg. (2022) 15:886–91. doi: 10.1136/jnis-2022-019202, PMID: 35853697 PMC10447392

[8] Molyneux AJ Ellison DW Morris J Byrne JV. Histological findings in Giant aneurysms treated with Guglielmi detachable coils: report of two cases with autopsy correlation. J Neurosurg. (1995) 83:129–32. doi: 10.3171/jns.1995.83.1.0129, PMID: 7782828

[9] Szikora I Turányi E Marosfoi M. Evolution of flow-diverter Endothelialization and Thrombus Organization in Giant Fusiform Aneurysms after flow diversion: a histopathologic study. Am J Neuroradiol. (2015) 36:1716–20. doi: 10.3174/ajnr.A4336, PMID: 26251428 PMC7968752

[10] Lee D Yuki I Murayama Y Chiang A Nishimura I Vinters HV. Thrombus organization and healing in the swine experimental aneurysm model. Part I. A histological and molecular analysis. J Neurosurg. (2007) 107:94–108. doi: 10.3171/JNS-07/07/0094 17639879

[11] Carneiro A Rane N Küker W Cellerini M Corkill R Byrne JV. Volume changes of extremely large and Giant intracranial aneurysms after treatment with flow diverter stents. Neuroradiology. (2014) 56:51–8. doi: 10.1007/s00234-013-1304-0, PMID: 24317754

[12] Sirakova K Penkov M Matanov S Minkin K Ninov K Hadzhiyanev A. Progressive volume reduction and long-term aneurysmal collapse following flow diversion treatment of Giant and symptomatic cerebral aneurysms. Front Neurol. (2022) 13:972599. doi: 10.3389/fneur.2022.972599, PMID: 36034286 PMC9403733

[13] Wang Z Tian Z Li W Wang J Zhu W Zhang M. Variation of mass effect after using a flow diverter with adjunctive coil embolization for symptomatic unruptured large and giant intracranial aneurysms. Front Neurol. (2019) 10:1–8. doi: 10.3389/fneur.2019.01191, PMID: 31798519 PMC6874129

[14] Sunohara T Imamura H Goto M Fukumitsu R Matsumoto S Fukui N. Neck location on the outer convexity is a predictor of incomplete occlusion in treatment with the pipeline embolization device: clinical and angiographic outcomes. Am J Neuroradiol. (2021) 42:119–25. doi: 10.3174/ajnr.A6859, PMID: 33184073 PMC7814796

[15] Oishi H Teranishi K Yatomi K Fujii T Yamamoto M Arai H. Flow diverter therapy using a pipeline embolization device for 100 Unruptured large and Giant internal carotid artery aneurysms in a single Center in a Japanese Population. Neurol Med Chir. (2018) 58:461–7. doi: 10.2176/nmc.oa.2018-0148, PMID: 30298832 PMC6236209

[16] Komatsu K Takagi Y Ishii A Kikuchi T Yamao Y Fushimi Y. Ruptured intranidal aneurysm of an arteriovenous malformation diagnosed by delay alternating with nutation for tailored excitation (DANTE)–prepared contrast-enhanced magnetic resonance imaging. Acta Neurochir. (2018) 160:2435–8. doi: 10.1007/s00701-018-3713-7, PMID: 30367252

[17] Oshima S Fushimi Y Okada T Nakajima S Yokota Y Shima A. Neuromelanin-sensitive magnetic resonance imaging using DANTE pulse. Mov Disord. (2021) 36:874–82. doi: 10.1002/mds.28417, PMID: 33314293 PMC8247273

[18] O’Kelly CJ Krings T Fiorella D Marotta TR. A novel grading scale for the angiographic assessment of intracranial aneurysms treated using flow diverting stents. Interv Neuroradiol. (2010) 16:133–7. doi: 10.1177/159101991001600204, PMID: 20642887 PMC3277972

[19] Raz E Goldman-Yassen A Derman A Derakhshani A Grinstead J. Vessel wall imaging with advanced flow suppression in the characterization of intracranial aneurysms following flow diversion with pipeline embolization device. J NeuroIntervent Surg. (2022) 14:1264–9. doi: 10.1136/neurintsurg-2021-018086, PMID: 34987073

[20] Shao Q Li Q Qiaowei W Li T Li L Chang K. Application of 3D T1-SPACE combined with 3D-TOF sequence for follow-up evaluation of stent-assisted coil embolization for intracranial aneurysm. J Intervent Med. (2021) 4:71–6. doi: 10.1016/j.jimed.2021.02.007, PMID: 34805951 PMC8562288

[21] T Fujii Teranishi K Yatomi K Suzuki K Mitome-Mishima Y Kondo A. Long-term follow-up results after flow diverter therapy using the pipeline embolization device for large or giant unruptured internal carotid artery aneurysms: single-center retrospective analysis in the Japanese population. Neurol Med Chir. (2021) 62:19–27. doi: 10.2176/nmc.oa.2021-0203, PMID: 34707069 PMC8754679

[22] Szikora I Marosfoi M Salomváry B Berentei Z Gubucz I. Resolution of mass effect and compression symptoms following endoluminal flow diversion for the treatment of intracranial aneurysms. Am J Neuroradiol. (2013) 34:935–9. doi: 10.3174/ajnr.A3547, PMID: 23493889 PMC7964645

[23] Slater LA Soufan C Holt M Chong W. Effect of flow diversion with silk on aneurysm size: a single center experience. Interv Neuroradiol. (2015) 21:12–8. doi: 10.1177/1591019915576433 PMC4757208 25934769

[24] Miyachi S Hiramatsu R Ohnishi H Yagi R Kuroiwa T. Usefulness of the pipeline embolic device for large and Giant carotid cavernous aneurysms. Neurointervention. (2017) 12:83–90. doi: 10.5469/neuroint.2017.12.2.83, PMID: 28955510 PMC5613049

[25] Patzig M Forbrig R Ertl L Brückmann H Fesl G. Intracranial aneurysms treated by flow-diverting stents: long-term follow-up with contrast-enhanced magnetic resonance angiography. Cardio Vascular Interv Radiol. (2017) 40:1713–22. doi: 10.1007/s00270-017-1732-z, PMID: 28685380

[26] Szikora I Seifert P Hanzely Z Kulcsar Z Berentei Z Marosfoi M. Histopathologic evaluation of aneurysms treated with Guglielmi detachable coils or matrix detachable microcoils. Am J Neuroradiol. (2006) 27:283–8. PMID: 16484393 PMC8148754

[27] Trivelato FP Ulhôa AC Rezende MT Castro-Afonso LH Abud DG. Republished: recurrence of a totally occluded aneurysm after treatment with a pipeline embolization device. J NeuroIntervent Surg. (2019) 11:E5. doi: 10.1136/neurintsurg-2018-013842.rep, PMID: 29794160

[28] Kulcsár Z Houdart E Bonafé A Parker G Millar J Goddard AJP. Intra-aneurysmal thrombosis as a possible cause of delayed aneurysm rupture after flow-diversion treatment. Am J Neuroradiol. (2011) 32:20–5. doi: 10.3174/ajnr.A2370, PMID: 21071538 PMC7964960

[29] Park MS Nanaszko M Sanborn MR Moon K Albuquerque FC McDougall CG. Re-treatment rates after treatment with the pipeline embolization device alone versus pipeline and coil embolization of cerebral aneurysms: a single-center experience. J Neurosurg. (2016) 125:137–44. doi: 10.3171/2015.7.JNS15582, PMID: 26684772

